# Wyoming State Medical Society

**Published:** 1898-12

**Authors:** E. Stuver

**Affiliations:** Secretary; Rawlins, Wyo.


*Editor Texas Medical Journal:*

### Society Notes.

### Wyoming State Medical Society.

RAWLINS, WYO., November 9, 1898.

*Editor Texas Medical Journal:*

The second regular meeting of the Wyoming State Medical Society, under the presidency of Dr. R. Harvey Reed, convened in Rock Springs November 1st. The meeting was a very profitable one. The papers were of a high order of merit, and elicited good discussions. Our society, though young, has put itself on record as being in favor of medical progress and correct ethical principles by adopting strong resolutions against the pending anti-vivisection bill (Senate Bill 1063), and by condemning the recent securing of a patent on diphtheria antitoxin by Prof. Behring. It also recommended the reorganization of the United States Army Medical Department on an independent basis, with the head of the department a cabinet officer.

The society was royally entertained by Drs. Freeman and R. Harvey Reed, and the citizens of Rock Springs. The next meeting will be held in Laramie City the second Tuesday in October, 1899.

E. STUVER, Secretary.

Officers for ensuing year: President, R. Harvey Reed, M. D.; Secretary and Editor, E. Stuver, M. D.; Treasurer, C. H. Solier, M. D.

